# Effects of STN and GPi Deep Brain Stimulation on Impulse Control Disorders and Dopamine Dysregulation Syndrome

**DOI:** 10.1371/journal.pone.0029768

**Published:** 2012-01-25

**Authors:** Sarah J. Moum, Catherine C. Price, Natlada Limotai, Genko Oyama, Herbert Ward, Charles Jacobson, Kelly D. Foote, Michael S. Okun

**Affiliations:** 1 Department of Neurology, Center for Movement Disorders & Neurorestoration, University of Florida, Gainesville, Florida, United States of America; 2 Department of Neurosurgery, Center for Movement Disorders & Neurorestoration, University of Florida, Gainesville, Florida, United States of America; 3 Deparatment of Clinical and Health Psychology, Center for Movement Disorders & Neurorestoration, University of Florida, Gainesville, Florida, United States of America; 4 Chulalongkorn Comprehensive Movement Disorders Center, Chulalongkorn University Hospital & Thai Red Cross Society, Bangkok, Thailand; 5 Department of Psychiatry, Center for Movement Disorders & Neurorestoration, University of Florida, Gainesville, Florida, United States of America; University Hospital La Paz, Spain

## Abstract

**Objective:**

Impulse control disorders (ICDs) and dopamine dysregulation syndrome (DDS) are important behavioral problems that affect a subpopulation of patients with Parkinson's disease (PD) and typically result in markedly diminished quality of life for patients and their caregivers. We aimed to investigate the effects of subthalamic nucleus (STN) and internal globus pallidus (GPi) deep brain stimulation (DBS) on ICD/DDS frequency and dopaminergic medication usage.

**Methods:**

A retrospective chart review was performed on 159 individuals who underwent unilateral or bilateral PD DBS surgery in either STN or GPi. According to published criteria, pre- and post-operative records were reviewed to categorize patients both pre- and post-operatively as having ICD, DDS, both ICD and DDS, or neither ICD nor DDS. Group differences in patient demographics, clinical presentations, levodopa equivalent dose (LED), and change in diagnosis following unilateral/bilateral by brain target (STN or GPi DBS placement) were examined.

**Results:**

28 patients met diagnostic criteria for ICD or DDS pre- or post-operatively. ICD or DDS classification did not differ by GPi or STN target stimulation. There was no change in DDS diagnosis after unilateral or bilateral stimulation. For ICD, diagnosis resolved in 2 of 7 individuals after unilateral or bilateral DBS. Post-operative development of these syndromes was significant; 17 patients developed ICD diagnoses post-operatively with 2 patients with pre-operative ICD developing DDS post-operatively.

**Conclusions:**

Unilateral or bilateral DBS did not significantly treat DDS or ICD in our sample, even though a few cases of ICD resolved post-operatively. Rather, our study provides preliminary evidence that DDS and ICD diagnoses may emerge following DBS surgery.

## Introduction

Dopamine agonist therapy and sometimes even levodopa therapy for Parkinson's disease (PD) may be associated with hypersexuality, pathological gambling, compulsive eating, compulsive shopping, and other ICDs [1]. In PD patients dopamine replacement therapy may also result in a pathological overusage of levodopa [2] and this condition has been termed the dopamine dysregulation syndrome (DDS) [3]–[5]. The effects of STN and/or GPi deep brain stimulation (DBS) on these issues remains largely unknown, however many groups have argued that DBS, particularly in the STN, may be beneficial for these syndromes by simply facilitating dopamine agonist and levodopa reduction [6], [7].

Most patients undergoing DBS are selected based on the potential for improvement of motor symptoms as well as for potential improvement in on-off medication fluctuations [8]–[10]. Optimal DBS candidates usually have excellent on-off dopaminergic responses documented by a dopamine challenge test. Patients with earlier onset PD have been observed to experience more severe motor fluctuations and to have a higher propensity to develop ICDs and DDS [11], [12]. These patients are more likely to be included in DBS cohorts [13].

The existing literature is undecided as to the optimal approach to treating patients with these debilitating behavioral disorders. We retrospectively reviewed our comprehensive patient database to report our experience with ICD and DDS and to specifically examine the effect of DBS on these disorders. Neurological, neurosurgical, neuropsychological, and psychiatric evaluations of each patient by an interdisciplinary DBS surgical board were carefully reviewed for ICD and DDS diagnostic criteria. The current investigation examined the effects of unilateral and bilateral DBS as well as lead placement (STN vs. GPi) on ICD/DDS group classification and change in dopaminergic medication usage for patients with idiopathic PD. The patient population was also studied to determine whether DBS might unmask these behavioral syndromes.

## Methods

### Ethics Statement

The reported study utilized a University of Florida Institutional Review Board (UF IRB) previously approved database for PD (INFORM-PD). The compiled data had been collected prospectively on all patients seen at the University of Florida Center for Movement Disorders & Neurorestoration. Written informed consent was received from all participants. To facilitate the current study, a second UF IRB approved retrospective chart review was conducted for all patients with PD identified using the database.

### Participants

An Institutional Review Board (IRB) approved retrospective chart review was performed on 159 patients who underwent unilateral or bilateral DBS surgery at the University of Florida Center for Movement Disorders & Neurorestoration between January 2002 and January 2010. All patients operated at the University of Florida underwent a complete in person evaluation with a neuropsychologist, a psychiatrist, a neurologist, and a neurosurgeon as part of the DBS screening process. The medical records from the screening process were reviewed as well as the records from the pre-operative interdisciplinary discussion (DBS board) and all post-operative follow-up visits (review included records from all four specialties).

Pre-operative and Post-operative Participant Classification – A detailed record review was utilized to identify any pre- or post-operative ICD or DDS. The diagnostic criteria used for DDS and specific ICDs are detailed in Table 1. DDS was defined according to Giovannoni's criteria [2]. Hypersexuality was defined according to diagnostic criteria proposed by Voon *et al.* [14]. Pathological gambling was defined using the DSM-IV definition [15]. Compulsive shopping was defined using McElroy's criteria [16]. Compulsive eating was defined according to the DSM-IV definition and Nirenberg *et al.* [15], [17]. Finally, punding was defined according to Friedman who initially observed punding behaviors, similar to those seen in individuals taking stimulants, among patients taking levodopa medication [18]. Records were excluded from analyses if DBS implantation occurred at an outside institution.

**Table 1:** Definitions of DDS, ICDs, and Punding.

**Dopamine Dysregulation Syndrome** *(Giovanni 2000)*		
A.	PD with documented levodopa responsiveness	
B.	Need for increasing doses of DRT in excess of those normally required to relieve Parkinsonian symptoms and signs	
C.	Pattern of pathological use: expressed need for increased DRT in the presence of excessive and significant dyskinesias despite being ‘on,’ drug hoarding or drug seeking behavior, unwillingness to reduce DRT, absence of painful dystonias	
D.	Impairment in social or occupational functioning: fights, violent behavior, loss of friends, absence from work, loss of job, legal difficulties, arguments or difficulties with family	
E.	Development of hypomanic, manic, or cyclothymic affective syndrome in relation to DRT	
F.	Development of a withdrawal state characterized by dysphoria, depression, irritability, and anxiety on reducing the level of DRT	
G.	Duration of disturbance of at least 6 months	
**Impulse Control Disorders**		
***Hypersexuality*** * (Voon 2006)*		
A.	The sexual thoughts/behaviors are excessive or an atypical change from baseline marked by 1 of the following:	
	1.	Maladaptive preoccupation with sexual thoughts
	2.	Inappropriately or excessively requesting sex from spouse or partner
	3.	Habitual promiscuity
	4.	Compulsive masturbation
	5.	Calls to telephone sex lines or viewing of pornography
	6.	Paraphilias
B.	The behavior must have persisted for at least 1 month	
C.	The behavior causes 1 of the following:	
	1.	Marked distress
	2.	Attempts to control thoughts or behavior that are unsuccessful or result in marked anxiety or distress
	3.	Becomes time consuming
	4.	Significant interference with social or occupational functioning
D.	The behavior does not occur exclusively during periods of hypomania or mania	
E.	If all criteria except C are fulfilled, the disorder is subsyndromal	
***Gambling*** * (DSM-IV)*		
A.	Persistent and recurrent maladaptive gambling behavior as indicated by 5 (or more) of the following:	
	1.	Preoccupation with gambling
	2.	Increasing amount of money wagered
	3.	Repeated unsuccessful attempts to control
	4.	Restlessness or irritability when cutting down
	5.	Gambles to escape from problems or to relieve dysphoric mood
	6.	Chases losses
	7.	Lies to others about gambling
	8.	Performs illegal acts to finance gambling
	9.	Jeopardized relationships, work, or education
	10.	Relies on others for money
B.	Does not occur exclusively during periods of hypomania or mania	
***Compulsive shopping*** * (McElroy 1994)*		
A.	Maladaptive preoccupation with buying or shopping that is manifested as impulses or behaviors	
	1.	Are experienced as irresistible, intrusive, and/or senseless
	2.	Result in frequent buying of more than can be afforded, of items that are not needed, or during longer periods of time than intended
B.	Cause marked distress, are time-consuming, significantly interfere with social or occupational functionoing, or result in financial problems	
C.	The behaviors do not occur exclusively during periods of hypomania or mania	
***Compulsive eating***		
*(DSM-IV)*		
A.	Recurrent episodes of binge eating, which is characterized by both of the following:	
	1.	Eating, in a discrete period of time (e.g. within any 2-hour period), an amount of food that is definitely larger than most people would eat in a similar period of time under similar circumstances
	2.	A sense of lack of control over eating during the episode (i.e. feeling that one cannot stop eating or control what or how much one is eating)
B.	The binge-eating episodes are associated with three (or more) of the following:	
	1.	Eating much more rapidly than normal
	2.	Eating until feeling uncomfortably full
	3.	Eating large amounts of food when not feeling physically hungry
	4.	Eating alone because of being embarrassed by how much one is eating
	5.	Feeling disgusted with oneself, depressed, or very guilty after overeating
C.	Marked distress regarding binge eating is present	
D.	The binge eating occurs, on average, at least 2 days a week for 6 months	
E.	The binge eating is not associated with the regular use of inappropriate compensatory behaviors (e.g. purging, fasting, excessive exercise) and does not occur exclusively during the course of Anorexia Nervosa or Bulimia Nervosa	
*(Nirenberg 2006)*		
A.	Uncontrollable consumption of a larger amount of food than normal in excess of that necessary to alleviate hunger	
**Punding** *(Friedman 1994)*		
A.	Stereotypical motor behavior in which an intense fascination with repetitive, purposeless movements, such as taking apart mechanical objects, handling, examining, and sorting common objects, or picking at oneself without stopping	

DDS; Dopamine dysregulation syndrome.

ICDs; Impulse control disorders.

Group Consensus – Two raters (S.J.M. and N.L.) served as the primary chart reviewers. They were required to review and be experts on the diagnostic criteria for ICDs and DDS prior to initiating the review process. For the study data, if raters disagreed on a diagnosis, discrepancies were resolved by tertiary expert raters to achieve consensus before conducting final analyses.

### DBS Implantation

The procedures were performed by a fellowship-trained neurosurgeon (K.D.F.). Multiple pass microelectrode mapping was conducted by a fellowship-trained neurologist/electrophysiologist (M.S.O.). The thresholds for stimulation-induced benefit and side effects were determined intra-operatively using macrostimulation via the implanted DBS lead. Each lead was placed in the sensorimotor region of the desired target (STN or GPi). Pulse generators were surgically implanted one month after lead placement, and patients were typically evaluated for programming and medication adjustments monthly for the first six months, and then every 3–6 months thereafter. Patients underwent unilateral DBS initially and were offered the addition of a contralateral DBS implantation after 6–7 months of follow-up if clinically indicated.

### Statistical Analyses

Patients were initially separated into those with unilateral DBS and those with bilateral DBS. In the preliminary analysis, patients were classified based on pre-operative diagnosis (i.e. ICDs, DDS, both DDS and ICDs, or no ICD or DDS). Diagnostic groups were compared on demographics and particularly gender, as it has been reported that men are predisposed to ICD and DDS [11], [12]. For both unilateral and bilateral DBS patients, the pre- and post-operative diagnoses were compared to determine whether the stimulation target (STN or GPi) had a significant effect on the outcome, whether any new diagnosis of ICD or DDS developed post-operatively, or whether symptoms of ICD or DDS resolved post-operatively. Analyses used non-parametric statistics (Kruskall-Wallace, Chi-Square). Significance was based on alpha of .05.

## Results

Of 159 participants, 24 patients (15.1%) met the diagnostic criteria for ICD and 7 (4.4%) for DDS, either pre- or post-operatively. Pre-operatively, 11/159 patients (6.9%) met criteria for an impulse control disorder or dopamine dysregulation syndrome (ICD = 6/159, 3.8%; DDS = 4/159, 2.5%; both ICD/DDS = 1/159, 0.6%).

### Diagnostic Group Demographics and Medication Usage

Table 2 shows baseline demographics and characteristics for all cohort patients. Pre-operative diagnostic subgroups were similar in age of onset for PD symptoms, age at the time of surgery, and motor score (UPDRS-III OFF MEDICATION, or UPDRS-III ON MEDICATION) (all *p*>0.05). There was no significant difference between diagnostic subgroups according to pre-operative dopamine agonist usage (χ^2^(6) = 6.51, ns), Hoehn and Yahr Staging OFF MEDICATION (χ^2^(15) = 9.51, ns), or Hoehn and Yahr Staging ON MEDICATION (χ^2^(12) = 3.94, ns). Regarding pre-operative levodopa equivalent dose (LED), there was similar LED usage between patients with ICD and DDS (Mean ± S.D.): ICD = 734.06 mg±388.32 mg, DDS = 1270.83mg±743.76 mg; Z = −1.03, ns) with the one ICD/DDS patient demonstrating LED usage that doubled that of the other groups (ICD/DDS = 2250.00 mg). Of the 159 patients in the cohort, 71.1% were male with all 5 pre-operative DDS patients were male. This gender difference, however, did not reach statistical significance (χ^2^(3) = 3.33, ns).

**Table 2:** Baseline Demographics and Characteristics for All Patients in the Cohort.

	ICD	DDS	ICD and DDS	No ICD or DDS	Total	Significance among subgroups
Mean age of PD (symptom) onset	44.20±7.40	45.00±1.00	40.00	48.84±9.54	48.54±9.40	F(3,141) = 0.82, *p* = 0.49
Mean age at time of surgery	58.50±7.50	60.50±7.14	50.00	61.61±8.86	61.39±8.77	F(3,155) = 0.82, *p* = 0.49
Mean UPDRS-III OFF MEDICATION	45.83±19.83	43.50±9.88	32.00	42.24±12.10	42.35±12.34	F(3,143) = 0.40, *p* = 0.75
Mean Hoehn and Yahr Staging OFF MEDICATION	3.00	3.00	2.5	2.87	2.88±0.71	χ^2^(15) = 9.51
Mean UPDRS-III ON MEDICATION	28.20±10.94	20.00±6.88	20.00	23.57±9.11	23.61±9.09	F(3,133) = 0.68, *p* = 0.56
Mean Hoehn and Yahr Staging ON MEDICATION	2.10	2.13	2.0	2.34	2.32±0.44	χ^2^(12) = 3.94
Mean pre-operative levodopa equivalent dose (mg)	734.06±388.32	1270.83±743.76	2250.00	877.11±510.88	888.80±522.32	F(3,141) = 3.13, *p* = 0.03
Pre-operative dopamine agonist usage	4/6	0/4	1/1	81/148	86/159	χ^2^(6) = 6.51
Gender (M/F)	3/3	4/0	1/0	105/43	113/46	χ^2^(3) = 3.33

MEDICATION; all PD medications.

### DDS and DBS Stimulation

Table 3 shows pre- and post-operative diagnoses for each patient with pre-operative DDS. After unilateral DBS placement, all 5/5 patients (100%) still fulfilled the DDS diagnostic criteria. Comparison of the effects of STN vs. GPi stimulation on DDS showed no change in DDS diagnosis relative to site of stimulation or pre-operative vs. 6 months post-unilateral DBS medication consumption according to LED level (all *p*>0.05).

**Table 3:** Did Unilateral and Bilateral DBS Lead Placement Impact the Pre-operative Diagnosis of DDS?

Patient	Pre-operative Diagnosis	Diagnosis after Unilateral DBS Placement* (Target)	Diagnosis after Bilateral DBS Placement* (Targets)
1	**DDS**	**DDS** (STN)	**
	Need for excessive DRT, frequent rescue doses, development of withdrawal state with dose reduction	Need for excessive DRT, frequent rescue doses, development of withdrawal state with dose reduction	
2	**DDS**	**DDS** (GPi)	**DDS** (GPi, GPi)
	Need for excessive DRT, self-medicating, development of withdrawal state with dose reduction	Need for excessive DRT, development of withdrawal state with dose reduction	Need for excessive DRT, development of withdrawal state with dose reduction
3	**DDS**	**DDS** (STN)	**DDS** (STN, STN)
	Need for excessive DRT, self-medicating, development of withdrawal state with dose reduction	Need for excessive DRT, self-medicating, development of withdrawal state with dose reduction, unwilling to reduce dosage	Need for excessive DRT, self-medicating, development of withdrawal state with dose reduction, unwilling to reduce dosage
4	**DDS**	**DDS** (STN)	**DDS** (STN, STN)
	Need for excessive DRT, unwilling to reduce dosage, development of withdrawal state with dose reduction	Need for excessive DRT, unwilling to reduce dosage, development of withdrawal state with dose reduction, increased need for DRT despite excessive dyskinesias	Need for excessive DRT despite being able to decrease dosage, increased need for DRT despite excessive dyskinesias
5	**ICD and DDS**	**ICD and DDS** (GPi)	**ICD and DDS** (GPi, GPi)
	Pathological gambling and shopping	Need for excessive DRT, excessive spending and gambling, excessive money spent on adult entertainment, cannot maintain finances	Need for excessive DRT, excessive spending and gambling, excessive money spent on adult entertainment, cannot maintain finances

*DBS unilateral or bilateral lead target(s) noted in parentheses.

**Patient did not have bilateral DBS lead placement.

DRT; dopamine replacement therapy including levodopa and/or dopamine agonists.

Of the 5 unilateral patients, 4/5 later had bilateral DBS placement. After bilateral DBS placement in the patients with pre-operative DDS, the diagnosis did not change (4/4 maintained DDS). Medication change after bilateral DBS could not be determined due to limited information as one patient died in an unrelated motor vehicle accident and another died of congestive heart failure (2/4 had LED information: preoperative = 1456.22 mg±1122.53 mg, 6 m post-bilateral = 1175.00 mg±35.36 mg). For these patients, 2/4 had bilateral placement in the STN and 2/4 patients had bilateral placement in the GPi.

### ICD and DBS Stimulation

Table 4 shows pre- and post-operative diagnoses for each patient with pre-operative ICD. Of the 7 patients with pre-operative ICDs, 1 patient underwent bilateral simultaneous DBS implantation and 6 patients underwent unilateral implantation. 4/6 patients went on to receive bilateral DBS placement. After unilateral DBS placement, 1/6 patients (16.7%) no longer met diagnostic criteria for ICD (placed in STN). Despite the resolution of ICD post-operatively for one patient implanted in the STN, the comparison of the effect of STN vs. GPi stimulation on ICD diagnosis did not yield a significant difference between the two targets (χ^2^(1) = 1.20, ns), although notably the sample size was small. Among the 6 unilateral DBS patients with pre-operative ICD, there also was no significant difference in pre-operative vs. 4 months post-unilateral DBS dopamine agonist usage (χ^2^(1) = 0.60, ns).

**Table 4:** Did Unilateral and Bilateral DBS Lead Placement Impact the Pre-operative Diagnosis of ICD?

Patient	Pre-operative Diagnosis	Diagnosis after Unilateral DBS Placement* (Target)	Diagnosis after Bilateral DBS Placement* (Targets)
1	**ICD**	**	**ICD and DDS** (STN, STN)
	Increased impulsivity, sexual indiscretions, punding		Increased impulsivity, sexual indiscretions, overmedicating with Sinemet, desire to increase dose unnecessarily
2	**ICD**	**ICD** (GPi)	**No ICD or DDS** (GPi, GPi)
	Excessive gambling and spending	Excessive gambling and spending	
3	**ICD**	**ICD** (GPi)	***
	Gambling, hyperphagia, compulsion to bake cakes	Gambling	
4	**ICD**	**ICD** (STN)	***
	Excessive chocolate cravings, hyperphagia	Increased sweet cravings, hyperphagia	
5	**ICD**	**ICD** (STN)	***
	Excessive gambling with scratch-off lottery tickets	Excessive spending, shopping, gambling with scratch-off lottery tickets	
6	**ICD**	**DDS** (STN)	**DDS** (STN, STN)
	Excessive gambling with scratch-off lottery tickets	Unwilling to lower dosage, development of withdrawal state with dose reduction	Unwilling to lower dosage, development of withdrawal state with dose reduction
7	**ICD and DDS**	**ICD and DDS** (GPi)	**ICD and DDS** (GPi, GPi)
	Pathological gambling and shopping	Need for excessive DRT, excessive spending and gambling, excessive money spent on adult entertainment, cannot maintain finances	Need for excessive DRT, excessive spending and gambling, excessive money spent on adult entertainment, cannot maintain finances

*DBS unilateral or bilateral lead target(s) noted in parentheses.

**Patient had simultaneous bilateral DBS lead placement. No diagnostic assessment was possible for the patient after unilateral placement.

***Patient did not have bilateral DBS lead placement.

DRT; dopamine replacement therapy including levodopa and/or dopamine agonists.

After bilateral placement, 2/4 patients (50%) with sufficient follow-up did not meet the diagnostic criteria for ICD. One case resolved after unilateral DBS and remained asymptomatic after the second surgery. The second case resolved after the additional contralateral DBS. There was no significant difference in pre-operative vs. 4 months post-bilateral DBS dopamine agonist usage (χ^2^(1) = 2.92, ns). Among the 2 patients with resolved ICD, one had bilateral placement in the STN, and the other had bilateral placement in the GPi.

### DBS Stimulation and Unmasking of Diagnoses

Among patients with pre-operative ICD, 2/7 subjects developed DDS after DBS placement, as shown in Table 4. Both of these patients were stimulated in the STN. Post-operative DDS was not diagnosed in any patient in our cohort who did not have either pre-operative DDS or ICD.

For ICD, among all of the DBS surgical interventions evaluated (unilateral and bilateral), 17 patients developed ICD post-operatively. Unilateral DBS was associated with 11/17 newly diagnosed ICDs. Of these 11 patients, 5 went on to receive bilateral DBS and in 4/5 patients (80%), the ICD resolved after the addition of contralateral DBS therapy. Staged bilateral DBS was associated with 6 new diagnoses of ICD after the second side procedure that had not been present after unilateral DBS. For all patients with new ICD diagnoses, there was no significant change in pre-operative vs. 4 months post-unilateral DBS dopamine agonist usage, pre-operative vs. 4 months post-bilateral DBS dopamine agonist usage, pre-operative vs. 6 months post-unilateral DBS LED level, or pre-operative vs. 6 months post-bilateral DBS LED level (all *p*>0.05). Of the patients who developed ICD post-unilateral DBS, 7/11 had lead placement in GPi and 4/11 had placement in STN. Of the patients who developed ICD post-bilateral DBS, 4/6 had bilateral placement in the STN and 2/6 had placement in the Gpi.

## Discussion

Using the criteria described above, we identified a group of 11 individuals with pre-operative DDS, ICD, or both ICD and DDS. Patients in this study all had interdisciplinary pre-operative evaluations for DBS, and these evaluations included questions addressing the criteria for diagnosing behavioral disorders. It is important however to note that when interpreting the data from this study, that the awareness of these behavioral issues evolved over many years, and therefore the reported numbers could represent an underestimate of these features.

The findings from this study suggest that unilateral or bilateral DBS had no clear effect on DDS, even if medication reduction was realized. Quantitating medication intake in DDS and ICD, however, can be challenging as exact doses and intervals may not be precisely known. The reason for the lack of precision is that inherent to these syndromes is the potential for taking multiple extra doses of medication. Moreover, since our center performs unilateral DBS frequently, results should be interpreted with caution, as bilateral DBS is associated with more medication reduction, and in a larger sample size may have led to greater improvement in these behavioral features.

GPi vs. STN stimulation targets had no appreciable differential effect on DDS symptoms. In contrast to the negligible effect of DBS on DDS symptoms, the effect of DBS on ICDs appeared more promising. One of seven patients with ICDs prior to surgery resolved their ICD after unilateral DBS placement and remained without ICD after staged bilateral DBS. An additional patient recovered from ICD after bilateral DBS. There was no significant decrease in dopamine agonist usage after either unilateral or bilateral DBS. This observation may be a result of our small sample size, however our results suggest that discontinuation of dopamine agonist usage was not completely responsible for ICD resolution. An important limitation of this study is that surgical target (STN vs. GPi DBS) was either determined by enrollment in a study (NIH COMPARE trial), or determined by interdisciplinary evaluation and expert discussion. This evaluative process may have led to a bias of implanting one target over another for various reasons including the potential for medication reduction with STN DBS. Future studies will need to include a randomized cohort.

DBS may have unmasked some behavioral issues for select patients, or alternatively DBS may have precipitated these behaviors. Prior to DBS surgery, 11 individuals had presentations consistent with ICDs, DDS, or both ICDs and DDS. Though often identified during the pre-operative assessment, the treatment of ICD and DDS has not been established, and as such groups performing DBS have not always specifically excluded these patients from surgery, and have also not routinely employed treatment programs. Data from this study would suggest it will be important in the future to identify and to address these issues pre-operatively if possible.

Interestingly, after either unilateral or bilateral DBS placement 2 patients displayed new diagnoses of DDS. Several patients who had not previously met the full diagnostic criteria for ICD or DDS met criteria for ICDs post-operatively. The emergence of post-DBS issues is an interesting and important observation [19], [20]. Potential explanations are that these behavioral issues were present, but not appreciated during pre-operative neuropsychological and psychiatric evaluations, or alternatively that DBS unmasked or precipitated the onset of these new symptoms. Larger, prospective studies will be necessary to clarify whether there is any causal relationship between DBS and the genesis or unmasking of these behavioral disorders, as has been shown in dopamine agonist use [1]. It should be considered that DBS may unmask underlying behavioral features, but also that DBS may, like medications, be capable of causing ICD or DDS. Another limitation of this study was that there was not a standardized approach to medication reduction post-DBS, and medication reduction may impact the appearance of these behavioral issues.

It is important to compare our cohort to the general PD literature. The DOMINION study reported a 13.6% ICD rate in the largest cohort reported to date [1]. In comparison, of 159 participants in our study, 24 patients (15.1%) met the diagnostic criteria for ICD and 7 (4.4%) for DDS, either pre- or post-operatively. Slight discrepancies in rate may be explained by differences in study designs, as well as differences between the two populations. Patients with ICD or DDS could potentially be excluded from DBS therapy, and therefore our numbers could also be considered an underestimate of the prevalence of these disorders. It will be important in the future to recruit a large multi-center cohort, such as was done for the DOMINION study, to further clarify DBS effects.

A comparison of the patient characteristics grouped by pre-operative diagnosis (i.e. ICD, DDS, both ICDs and DDS, or no ICD or DDS) demonstrated that there was no significant difference between the pre-operative diagnostic groups based on age of onset of PD, age at surgery, duration of symptoms prior to surgery, UPDRS-III OFF MEDICATION, UPDRS-III ON MEDICATION, pre-operative dopamine agonist usage, Hoehn and Yahr Staging OFF MEDICATION, or Hoehn and Yahr Staging ON MEDICATION. Although the DDS patients had a higher mean pre-operative medication consumption relative to the ICD group, this difference was not significant. These observations are consistent with the body of literature on this subject [12]. Also, in the DDS pre-operative group, we observed that all of the patients within the DDS subgroup were male. This observation suggests a strong predisposition to DDS among men and is supported by previous literature [12]. A male predominance has recently also been suggested for certain subtypes of ICDs [11].

The previously unappreciated prevalence of these Parkinson-related behavioral disorders and the profound detrimental effect that they have on the quality of life of patients and their caregivers have resulted in a new appreciation among clinicians and researchers of the critical need for an effective treatment for ICD and DDS. Unfortunately, our understanding of these disorders is limited and their clinical management remains quite challenging. Decreasing or completely discontinuing a patients' dopaminergic medications may reduce the impulsivity observed in ICDs and may also aid in mitigating the pathological medication usage seen in DDS. This approach, however, predictably exacerbates Parkinsonian motor symptoms [21]. When these measures fail, psychotherapeutic interventions may potentially be implemented with some symptomatic improvement. Cognitive behavioral therapy has also been shown to have some efficacy in treating ICDs, such as pathological gambling [22]. Objective research on the efficacy of psychiatric drugs, particularly for ICDs, is limited [21]. Selective reuptake inhibitors and neuroleptics such as clozapine and risperidone have been shown to help control ICDs [23]–[25], while valproic acid and lithium have been shown to improve ICDs on a more case-specific basis [26], [27]. Larger studies are needed to verify these potential pharmacologic effects.

Clinicians have been recently interested in studying the effects of DBS on ICDs and/or DDS, especially since these entities have proven to be difficult to address pharmacologically [7], [19], [20], [28]–[30]. Some studies suggest that DBS improves DDS, while others indicate that DBS has no effect [7]. To complicate matters, while some studies indicate that DBS may be an effective therapy for ICDs, others suggest that it promotes their development [19], [20], [28], [29]. All of these previously published studies included only a handful of patients. Well-designed, prospective studies will be required to elucidate the true effects of stimulation in various basal ganglia targets and to determine whether DBS improves, unmasks existing, or precipitates new DDS or ICDs. We suspect, based on the current analysis, that the story is complex and will require a much larger sample size to adequately sort out.

In conclusion, our experience indicated that neither unilateral nor bilateral DBS in the STN or the GPi resulted in resolution of pre-operative dopamine dysregulation syndrome. DBS did, however, show a potential therapeutic effect in two patients with impulse control disorders. One important observation was that DBS appeared to unmask or alternatively precipitate ICDs in some patients. It will be important in future studies to address the mechanisms that may underpin DBS precipitating these disorders. It will need to be determined whether direct limbic stimulation, or alternatively DBS is providing a second hit to a vulnerable genetic background or other process. Screening paradigms for DBS may need to be enhanced to include impulsivity, gambling, and other behavioral measures. Finally, family and spousal input seem to be important in uncovering at risk individuals. Careful prospective screening for these disorders and larger, prospective multi-center studies will be necessary to clarify the effects of DBS on DDS and ICDs. This research should address both therapeutic and potentially deleterious effects of DBS on these disorders.

Based on currently available evidence, clinicians should not consider unilateral or bilateral STN or GPi DBS to be a solution to Parkinsonian ICDs or DDS. Rather, heightened sensitivity to the significant prevalence and profound impact of these Parkinson-related behavioral disorders is warranted along with a more comprehensive approach to pre- and post-operative care. Patients should be carefully screened for ICDs and DDS before and after surgery to assess the true impact of DBS on these disorders. A thoughtful, patient-tailored treatment strategy for ICDs and DDS may also include judicious reduction of dopaminergic medications and behavioral therapies.
